# Exogenous phosphatidylglucoside alleviates cognitive impairment by improvement of neuroinflammation, and neurotrophin signaling

**DOI:** 10.1002/ctm2.332

**Published:** 2021-03-26

**Authors:** Yanjun Liu, Junyi Liu, Peixu Cong, Tao Zhang, Changhu Xue, Jie Xu, Yuming Wang, Xiangzhao Mao, Jingfeng Wang

**Affiliations:** ^1^ College of Food Science and Engineering Ocean University of China Qingdao Shandong Province China; ^2^ School of Food Science and Technology Jiangnan University Wuxi Jiangsu Province China; ^3^ Laboratory of Marine Drugs and Biological Products Pilot National Laboratory for Marine Science and Technology (Qingdao) Qingdao China; ^4^ College of Food Science and Engineering Nanjing University of Finance and Economics Nanjing Jiangsu China

Dear Editor,

Alzheimer's disease (AD) is a commonly progressive disorder of neurodegenerative disease.^1^ Until now, no approach to treat the pathological progression of AD has been proven to be effective.^1^ Our data favored that the treatment of phosphatidylglucoside (PtdGlc), a novel glucosylated lipid enriched in the brain, protects against Aβ and tau pathology, cognition deficits in APP/PS1 mice, and alleviates neuroinflammation through activation of PPARγ and restoration of the neurotrophin signaling. Our findings offers new perspectives for PtdGlc in the management of AD.

To date, the knowledge on the biology function of PtdGlc is limited and the impact of PtdGlc on the course of AD has never been studied. Based on the previous studies, PtdGlc appears to play an important role for brain development.^2^, ^3^, ^4^, ^5^, ^6^ Initially, we hypothesized that PtdGlc can aggravated the development of AD, due to the negative effect of lysoPtdGlc (hydrolytic derivative of PtdGlc) mediated nociceptive afferent axons in the central nervous system.^6^ And interestingly enough, our results strongly suggest that PtdGlc supplementation could be considered as a therapeutic strategy for AD. All of these results are likely to have important implications in the prevention and treatment with PtdGlc on AD, and there are no such data for its mechanism presently.

To demonstrate the role of PtdGlc on cognitive impairment, we synthesized PtdGlc and its analogues (phosphatidylfructoside [PtdFru], phosphatidylgalactoside [PtdGal], and phosphatidylriboside [PtdRib]) from soybean phosphatidylcholine by a phospholipase D (PLD)‐mediated transphosphatidylation (Figure S1). In this study, 1‐phosphatidyl‐β‐D‐glucose (1‐PtdGlc) comprised C16:0 and C18:1 was used (Figures S2 and S3). To examine whether the PtdGlc reached to brain, the mice was injected with stable isotope‐labeled PtdGlc (^13^C6‐D‐PtdGlc) via the tail vein. Of note, isotope‐labeled PtdGlc was observed in the brain of ^13^C6‐PtdGlc treated mice, which demonstrated that PtdGlc could reach to brain (Figure S4).

The effect of PtdGlc and its analogues on cognitive function was evaluated first. The 20‐week age male wild‐type and APPswe/PS1dE9 (APP/PS1) mice were randomly allocated to six groups: WT, APP/PS1; APP/PS1 supplemented with 0.1% PtdGlc, PtdFru, PtdGal, or PtdRib (Table S1). After 16 weeks of daily treatment, behavioral tests were carried out. Compared to APP/PS1 mice, PtdGlc, PtdGal, and PtdRib treatment decreased escape latency progressively from day 1 to 5, increased platform area crossings numbers and the percentage of time in the target quadrant at day 5 (Figures 1A‐C), but not swimming speed (Figure 1D). Only the mice receiving PtdGlc diet manifested a significant increase of the entries numbers (Figure 1E), and showed preference for a spatial strategy (Figures 1F, G). Consistent with the result of Morris water maze test, PtdGlc treatment showed improved reference and working memory acquisition compared to controls, suggesting improved spatial memory formation (Figures S5A, B). Studies have shown both of Aβ plaques and tau tangles pathology are key features of AD.^7^ Immunohistochemistry and immunofluorescence staining of Aβ indicated that PtdGlc treatment reduced the Aβ‐positive plaques in the cortex and hippocampus compared to those of untreated mice (Figures 1H, I). Elisa analysis data further confirmed this result (Figures S6A, D). We found that PtdGlc reduced BACE1 and Nicastrin levels in the hippocampus of AD mice (Figures S6B, C), which suggested that PtdGlc inhibits APP cleavage. Comparing to AD mice, PtdGlc treatment alleviated the expression of total Tau and phosphorylation of tau in hippocampus, but not the levels of p‐GSK3β (Figures 2E, F). As shown in Figure S7, we found that neurofibrillary tangles appeared obviously in AD mice, but not in the mice with the treatment of PtdGlc, PtdRib, or PtdGal, as judged by a Bielschowsky silver staining. Histopathological analysis (Figure 1J) confirmed a marked decrease in Tau protein expression of the hippocampus in PtdGlc‐fed mice as well as the mice treated with PtdFru, PtdGal, and PtdRib.

**FIGURE 1:**
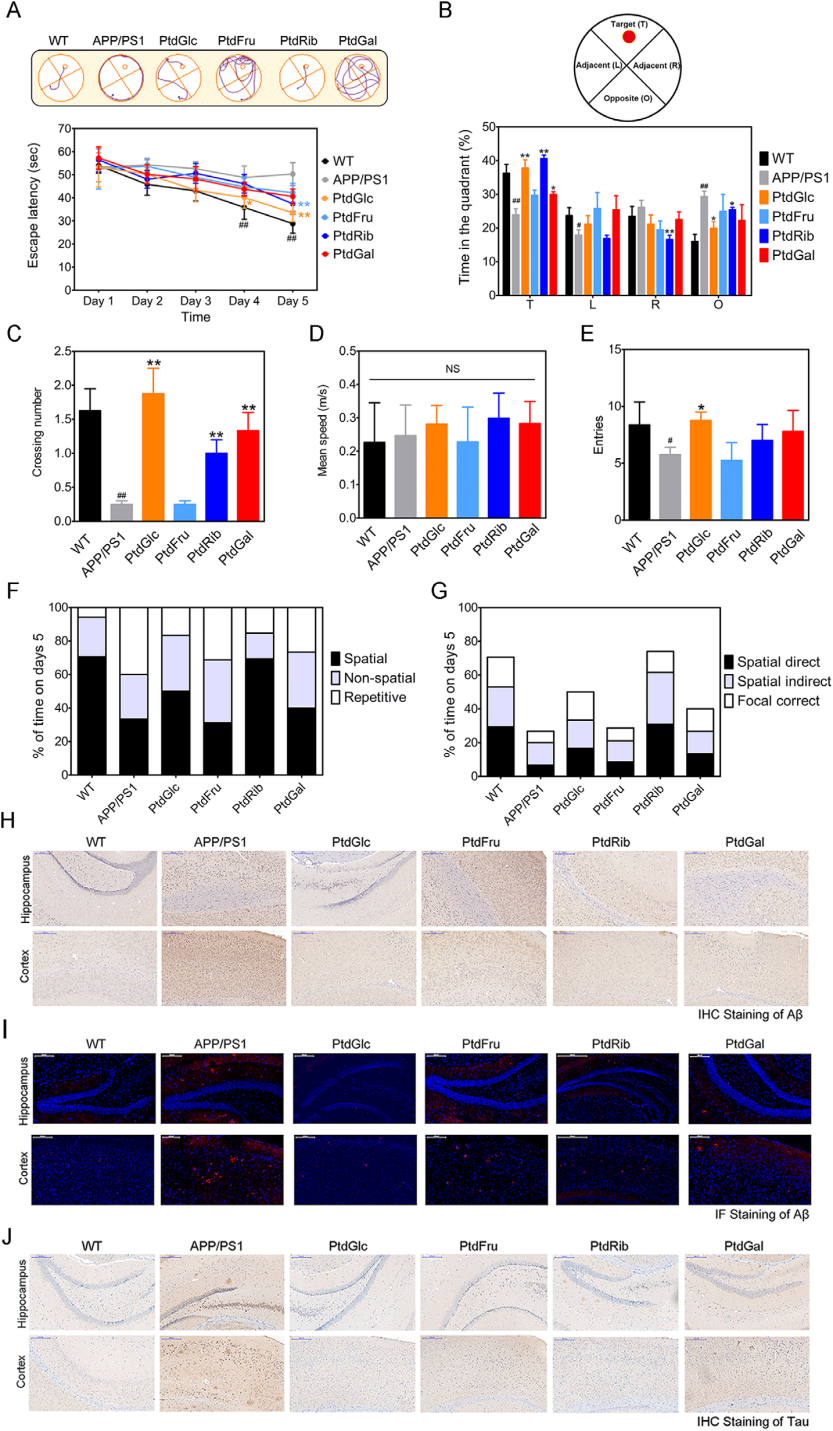
PtdGlc supplementation ameliorated memory deficits, reduced Aβ and Tau pathology. (A) Escape latency to the platform during the training trails in a Morris water maze and representative track images of mice (*n* = 5 mice/group). (B) Time spent in target quadrant in the MWM test at day 5. (C) Times crossing the target sites after retrieval of the platform at day 5. (D) Entries in target quadrant. (E) Average speed to find the platform. (F) Assessment of search strategy (spatial, nonspatial, repetitive) in the acquisition phase of the water maze test. (G) The percentage of time engaged in spatial search strategies during the 60‐second trial was calculated, with search strategies combined into 3 groups based on functional similarity (Spatial Direct, Spatial Indirect, and Focal Correct strategies). (H, I) Photomicrographs of Aβ plaques within the hippocampus of APP/PS1 and PtdGlc‐fed mice (*n* = 6 mice/group). Scale bar, 200 μm. (J) IHC of tau. Scale bar, 200 μm. Statistical significance was determined by Student's *t* test, *P < .05, **P < .01 vs APP/PS1 mice; #*P* < .05, ##*P* < .01 vs WT mice

**FIGURE 2:**
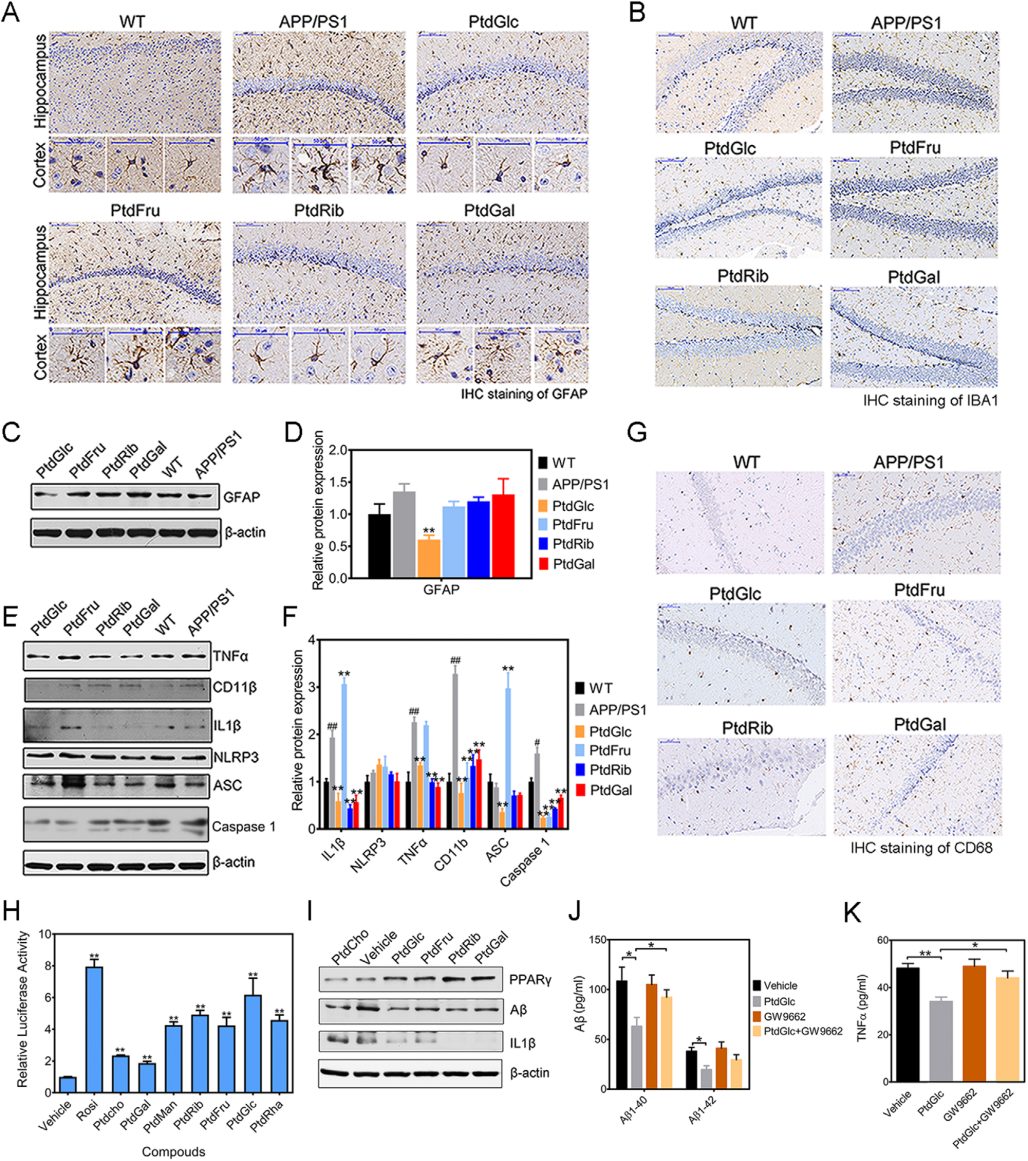
PtdGlc supplementation ameliorates neuroinflammation and reduce Aβ levels through PPARγ. (A) Photomicrographs depicting the hippocampus and cortex from APP/PS1 mice (*n* = 6 mice/group) stained for GFAP. Scale bar, 200 μm (Top) and 50 μm (bottom). (B) Photomicrographs depicting the hippocampus from APP/PS1 mice (*n* = 6 mice/group) stained for Iba1. Scale bar, 200 μm. (C, D) Representative western blot indicating the expression of GFAP in the hippocampus of APP/PS1 mice (*n* = 6 mice/group). (E, F) Representative western blot indicating the expression of neuroinflammation markers in the hippocampus of APP/PS1 mice (*n* = 6 mice/group). (G) Photomicrographs depicting the hippocampus from APP/PS1 mice (*n* = 6 mice/group) stained for CD68. Scale bar, 200 μm. (H) NIH3T3 cells were transfected with PPARγ expression vector and a luciferase reporter and treated with PtdGlc and its analogues (*n* = 3). (I) Representative western blot indicating the expression of PPARγ, Aβ, and IL1β in SH‐SY5Y_APP595/596_ cells treated with PtdGlc and its analogues (*n* = 5). (J, K) Levels of Aβ and TNFα was examined by Elisa in the all groups (Vehicle, GW9662, PtdGlc+GW9662 and PtdGlc‐treated groups), *n* = 5/group. Statistical significance was determined by Student's *t* test, *P < .05, **P < .01 as indicated

Next, the effect of PtdGlc on neuroinflammation was investigated. As indicated by staining for the activated astrocytes marker, GFAP, and activated microglia marker, Iba1, PtdGlc treatment reduced reactive astrocytes and microglia compared with APP/PS1 mice (Figures 2A, B). Supporting the above data, GFAP protein expression level was significantly decreased by PtdGlc (Figures 2C, D). Activated microglia cells and astrocytes can trigger inflammatory processes and regulate neuroinflammation in the brain.^8^ ASC and Caspase 1 levels were reduced by PtdGlc treatment in APP/PS1 mice, while NLRP3 expression was unchanged. Regarding the expression of TNFα and IL‐1β and the number of CD68‐immunopositive cells, there was a significant decrease in PtdGlc and other phosphatidyl saccharides except PtdFru, compared to the APP/PS1 mice (Figures 2E‐G). Based on a PPARγ‐dependent reporter assay, all phosphatidyl saccharides showed PPARγ agonist activity, with PtdGlc being the most potent and efficacious (Figure 2H). In a SH‐SY5Y_APP595/596_ cell line that expresses APP gene with 595/596 mutation, PtdGlc promoted protein expression of PPARγ remarkably. In contrast to PPARγ activation, Aβ protein expression was significantly decreased by PtdGlc (Figure 2I). Furthermore, treatment with PtdGlc, PtdRib, and PtdGal reduced IL1β protein expression and TNFα levels (Figures 2J, K). Next, Elisa analysis was performed to explore the effects of PtdGlc on Aβ levels in SH‐SY5Y_APP595/596_ cells and test whether the PPARγ antagonist block the beneficial effect of PtdGlc on inflammatory cytokines and Aβ production. Aβ1‐40 and Aβ1‐42 levels after PtdGlc treatment were lower than those in the untreated group. Of note, the effect on Aβ1‐40 and Aβ1‐42 was antagonized by GW9662 (PPARγ antagonist). Moreover, consistent with this result, pretreatment of cells with PtdGlc markedly attenuated the secretion of TNFα, whereas GW9662 almost abolishes this effect. These findings demonstrate that PPARγ is involved in the anti‐neuroinflammatory and anti‐Aβ effects of PtdGlc.

Although we now know PtdGlc appears to attenuate cognitive deficits, the growth of knowledge about the role of PtdGlc in the specific role of neuron functions is still limited. We found PtdGlc treatment increased immunoreactivity to neuronal marker NeuN and MAP‐2 (Figure 3A). Golgi staining results (Figure 3B) showed that PtdGlc‐treated mice showed the increase in dendritic branches and spine density (Figures 3C, D). To find out whether neurotrophins signaling were altered with or without PtdGlc treatment, we tested the neurotrophic factors levels and protein expressions of their receptors. Phosphorylated TrkA were markedly activated after the treatment with PtdGlc, while p75NTR, another NGF receptor, were inhibited by PtdGlc and PtdFru supplementation (Figures 3E, F). In addition, supplementation of PtdGlc increased the expression of NGF as assessed by IHC staining compared with AD mice (Figure 3H). Of note, significant increases in BDNF and phospho‐TrkB levels, but not TrkB, in the hippocampus of PtdGlc‐fed mice (Figures 3E‐G). The above results indicated that PtdGlc rescued NGF/TrkA signaling deficits as well as BDNF‐TrkB signaling, and inhibit the activation of p75NTR signaling. Compared with APP/PS1 mice (Figure 3I), the fractional areas stained for NeuN (Neuronal nuclei)‐ and Casp3 (caspase 3)‐positive axons were increased in PtdGlc‐, PtdRib‐, and PtdGal‐ treated groups. Consistent with these results, the expression of Bcl2 were all increased after PtdGlc treatment and caspase 3 and caspase 9 levels of were reduced after PtdGlc treatment, and the number of TUNEL positive cells was reduced after PtdGlc (Figures 3J‐L) compared with the mice in APP/PS1 group, indicating that the PtdGlc suppressed the neuronal apoptosis in AD.

**FIGURE 3:**
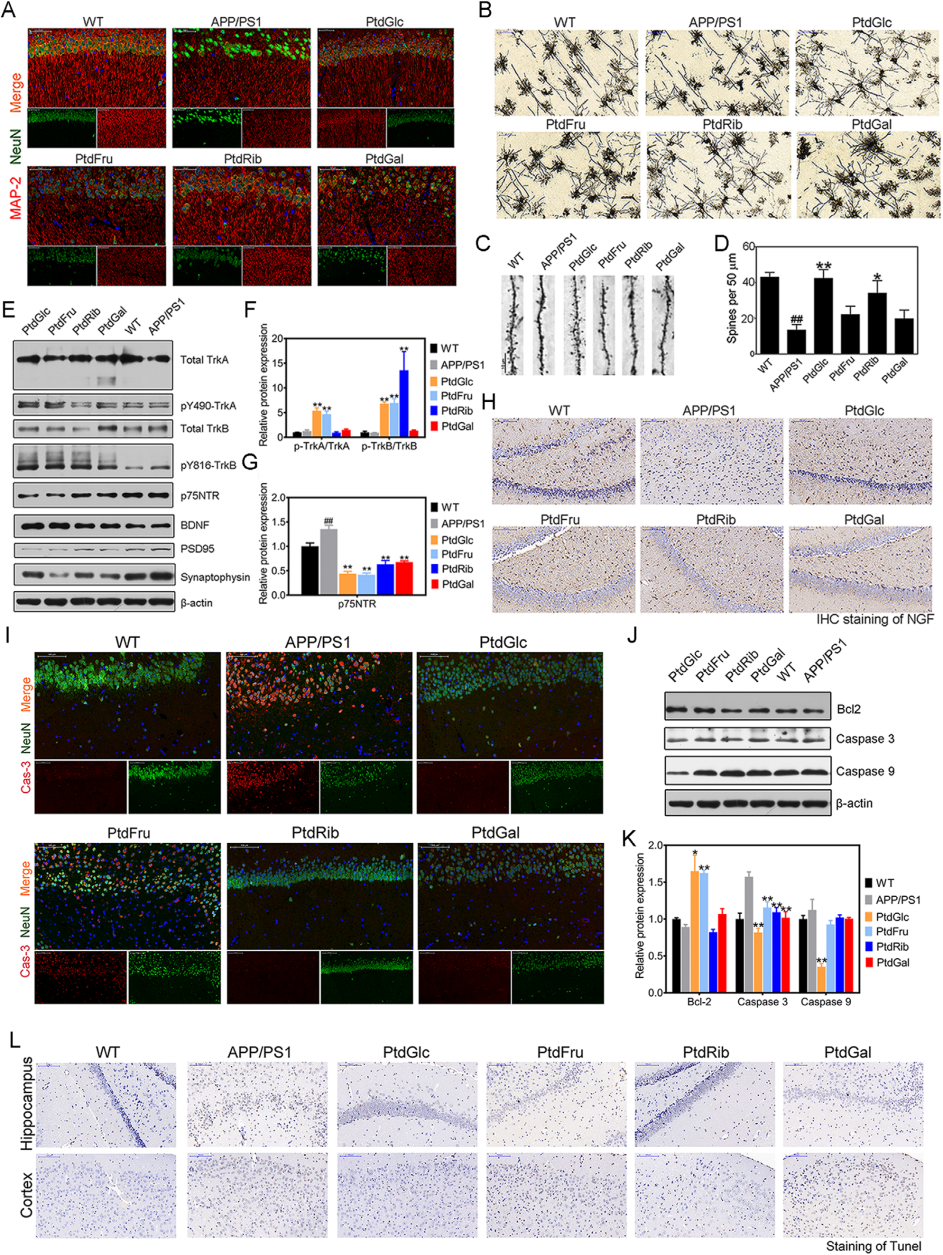
PtdGlc supplementation restores neurotrophin signaling and ameliorates neurodegeneration and neuronal death in APP/PS1 mice. (A) Photomicrographs depicting the hippocampus from APP/PS1 mice (*n* = 6 mice/group) stained for MAP‐2 (Red) and NeuN (Green). Scale bar, 50 μm. (B) Golgi staining was conducted on brain sections from CA1 region. Scale bar, 50 μm. (C) Dendritic spine and (D) its density was conducted by Golgi staining measured then (*n* = 9 sections from 3 mice in each group). (E, F, G) Representative western blot indicating the expression of neurotrophins and their receptors in the hippocampus of APP/PS1 mice (*n* = 6 mice/group). (H) Photomicrographs depicting the hippocampus from APP/PS1 mice (*n *= 6 mice/group) stained for NGF. Scale bar, 200 μm. (I) Photomicrographs depicting the hippocampus from APP/PS1 mice (*n* = 6 mice/group) stained for Cas3 (Red) and NeuN (Green). Scale bar, 50 μm. (J, K) Representative western blot indicating the expression of apoptosis‐related markers in the hippocampus of APP/PS1 mice (*n* = 6 mice/group). (L) Photomicrographs depicting the hippocampus from APP/PS1 mice (*n* = 6 mice/group) stained for TUNEL. Scale bar, 200 μm. Statistical significance was determined by Student's *t* test, *P < .05, **P < .01 vs APP/PS1 mice; #*P* < .05, ##*P* < .01 vs WT mice

In conclusion, current findings indicate that a dietary PtdGlc attenuate cognitive deficits. PtdGlc treatment reduced Aβ production and hippocampal neuroinflammation, which is likely through the activation of the PPARγ. Remarkably, we also found PtdGlc benefits on reducing neurogeneration and attenuating synaptic plasticity, maintaining neurotrophin signaling. Overall, the intervention of PtdGlc may provide a potential therapeutic agent or an approach to counter Alzheimer's Disease.

## CONFLICT OF INTEREST

The authors declare that they have no conflict of interest.

## Supporting information

Supporting information

## References

[1] Rodríguez‐Gómez O , Palacio‐Lacambra ME , Palasí A , Ruiz‐Laza A , Boada‐Rovira M . Prevention of Alzheimer's disease: a global challenge for next generation neuroscientists. J Alzheimers Dis. 2014;42:515‐523. 10.3233/JAD-141479 25351111

[2] Murate M , Hayakawa T , Ishii K , et al. Phosphatidylglucoside forms specific lipid domains on the outer leaflet of the plasma membrane. Biochemistry. 2010;49(23):4732‐4739. 20433166 10.1021/bi100007u

[3] Kinoshita MO , Furuya S , Ito S , et al. Lipid rafts enriched in phosphatidylglucoside direct astroglial differentiation by regulating tyrosine kinase activity of epidermal growth factor receptors. Biochem J. 2009;419(3):565‐575. 19170657 10.1042/BJ20081896

[4] Nagatsuka Y , Horibata Y , Yamazaki Y , et al. Phosphatidylglucoside exists as a single molecular species with saturated fatty acyl chains in developing astroglial membrane. Biochemistry. 2006;45(29):8742‐8750. 16846217 10.1021/bi0606546

[5] Nagatsuka Y , Hara‐Yokoyama M , Kasama T , et al. Carbohydrate‐dependent signaling from the phosphatidylglucoside‐based microdomain induces granulocytic differentiation of HL60 cells. P Natl Acad Sci USA. 2003;100(13):7454‐7459. 10.1073/pnas.1232503100 PMC164607 12802014

[6] Guy AT , Nagatsuka Y , Ooashi N , et al. Glycerophospholipid regulation of modality‐specific sensory axon guidance in the spinal cord. Science. 2015;349(6251):974‐977. 26315437 10.1126/science.aab3516

[7] Bassil F , Brown HJ , Pattabhiraman S , et al. Amyloid‐beta (Aβ) plaques promote seeding and spreading of alpha‐synuclein and tau in a mouse model of lewy body disorders with Aβ pathology. Neuron. 2020;105(2):260‐275. 31759806 10.1016/j.neuron.2019.10.010 PMC6981053

[8] Bouvier DS , Murai KK . Synergistic actions of microglia and astrocytes in the progression of Alzheimer's disease. J Alzheimers Dis. 2015;45(4):1001‐1014. 25663025 10.3233/JAD-143156

