# Supplementary material for: Exogenous phosphatidylglucoside alleviates cognitive impairment by improvement of neuroinflammation, and neurotrophin signaling
Source: Clin Transl Med. 2021 Mar 26;11(3):e332. doi: 10.1002/ctm2.332 (PMC8002902; doi:10.1002/ctm2.332)
Supplement: Supplementary file 1 — Supporting information [file CTM2-11-e332-s001.doc]

**Exogenous phosphatidylglucoside alleviates cognitive impairment by improvement of neuroinflammation, and neurotrophin signaling**

Yanjun Liu,^1,3^ Junyi Liu,^1^ Peixu Cong,^1^ Tao Zhang,^4^ Changhu Xue,^1,2^,* Jie Xu,^1^,* Yuming Wang,^1^ Xiangzhao Mao,^1,2^,* Jingfeng Wang^1^

^1^ *College of Food Science and Engineering, Ocean University of China, 5 Yushan Road, 266003 Qingdao, Shandong Province, China.*

^2^ *Laboratory of Marine Drugs and Biological Products, Pilot National Laboratory for Marine Science and Technology (Qingdao), Qingdao 266237, China*

^3^ *School of Food Science and Technology, Jiangnan University, 1800, Lihu Road, 214122, Wuxi, Jiangsu Province, China.*

^4^ *College of Food Science and Engineering, Nanjing University of Finance and Economics, Nanjing 210023, Jiangsu, China*

^*^ Corresponding author. Tel.: +8653282032468; fax: +86 532 82032408. E-mail address: xuechanghuouc@163.com (Changhu Xue).

^*^ Corresponding author. Tel.: +8653282031908; fax: +86 532 82031908. E-mail address: xujie9@ouc.edu.cn (Jie Xu).

^*^ Corresponding author. Tel.: +8653282032660; fax: +86 532 82032660. E-mail address: xzhmao@ouc.edu.cn (Xiangzhao Mao).

**Supplemental materials and methods**

**Preparation of several phospholipids**

PtdCho was prepared from soybean, and the fatty acid composition was palmitic plus stearic acids (16:0 and 18:0). PtdGlc, PtdFru, PtdMan, PtdGal, PtdRha and PtdRib were produced by transphospatidylation from PtdCho. Brifely, PtdCho were dissolved in ethanol. Silica of the same weight was added to the solution and mixed and the ethanol was removed (rotary evaporation). The reaction was carried out in 50-mM acetic acid–sodium acetate buffer (pH=7.0) with glucose and PLD (32 U/mL) at 40°C and stirred with a magnetic stirrer (600 rpm) for 8 hours. After that, chloroform/methanol (2:1, v/v) was added to isolate the PtdGlc.

**Animals models and diets**

The protocols were carried out in accordance with the ARRIVE Guide for the Care and Use of Laboratory Animals and approved by the Animal Ethics Committee of the Ocean University of China. 20 weeks-old male APPswe/PS1dE9 transgenic mice in a C57BL/6J background and littermate wild-type (WT) mice were purchased from the Model Animal Research Center of Nanjing University, China. Mice were maintained under a 12/12h (light/dark) at 22-26 °C with 60 ± 10% humidity. The PtdGlc group was fed with normal diet containing 0.1% PtdGlc. The composition of experimental diets was shown in the [supplemental Table](https://www.sciencedirect.com/science/article/pii/S1756464617307405#s0170) 1.

**Cell culture**

Human neuroblastoma cell line SH-SY5Y_APP595/596_ (a SH-SY5Y cell line that stably expresses APP gene with 595/596 mutation) was cultured in DMEM growth medium (10% fetal bovine serum). SH-SY5Y_APP595/596_ cells were treated with PtdGlc (200 ug/ml), or GW9662 alone (10 μM) or PtdGlc (200 ug/ml) combined with GW9662 (10 μM). After 24h, cell lysates were collected.

**Cell-based luciferase reporter gene assay**

PPARγ-dependent reporter assay was performed as reported previously. NIH3T3 cells were transfected with the pGL3B-Luc reporter plasmids (with promoter region of PPARγ) for 24 hours before treatment with rosiglitazone (Rosi, 1 μM), several phospholipids (PtdFru, PtdMan, PtdGal, PtdRha and PtdRib, 200 ug/ml) for 24 h.

**Analysis of several phospholipids**

The products after PLD mediated reaction were analyzed by TLC developed with chloroform/methanol/0.8 M NH_4_OH (75:25:3) and HPLC-MS/MS. The lipid species composition and the corresponding content of lipid were determined by a 6410 triple-quadrupole mass spectrometer equipped with an electrospray ion source (Agilent Technologies, USA). The lipid solutions were analyzed using a methanol mixture (1:1, v/v) as the carrying solvent at a flow rate of 0.15 mL/min. The TurboIon-Spray interface was manipulated in positive and negative ionization modes. Source parameters were as follows: capillary voltage, 5.5 kV (+ESI) and 4.5 kV (-ESI); temperature, 350°C; nebulizer pressure (N_2_), 25 (−ESI) and 35 (+ESI).

**Preparation and analysis of ^13^C6-D-PtdGlc**

^13^C6-D-PtdGlc were produced by phospholipase D mediated transphosphatidylation from PtdCho. Briefly, the PLD-catalyzed transphosphatidylation reaction between PtdCho and ^13^C6-D-Glucose was carried out using a glass vial with screw caps. PtdCho were dissolved in ethanol. Silica of the same weight was added to the solution and mixed for 30 min at room temperature. After that, the ethanol was removed by rotary evaporation. The reaction was carried out in 50-mM acetic acid–sodium acetate buffer (pH=7.0) with glucose and PLD (32 U/mL) at 40°C and stirred with a magnetic stirrer (600 rpm) for 8 hours. Further solid phase extraction purification and gradient elution was then carried out, including the washing conditions of 10ml of chloroform, 5ml of chloroform/methanol (9/1, v/v), 3ml of chloroform/methanol (2/1, v/v), 5ml of chloroform/methanol (1/2, v/v), and 5ml methanol.

**Morris water maze test**

A circular pool was filled with water made opaque by milk addition and divided into four quadrants. A black escape platform was located in the center of one quadrant and the mice were subjected to acquisition trial a day for 5 days. The mice were manually guided to the platform, when the mice were failed to locate the platform within 60 s. At last day, the mice were allowed to swim in the pool for 60 s and the number crossing over the previous position of the platform and the time spent in the target quadrant were recorded.

**Silver staining**

NFTs were detected by Bielschowsky silver staining method. Briefly, Brain sections were placed in 10% silver nitrate solution for 15 minutes and then placed in ammonium silver nitrate solution at 40°C for 30 minutes. Sections were placed in 1% ammonium hydroxide solution to stop the reaction.

**Immunohistochemistry and immunofluorescence staining**

Mice were euthanized and systemically perfused with phosphate-buffered saline (PBS) and 4% paraformaldehyde solution, then the brain samples were collected and fixed in 4% paraformaldehyde solution for 48h at 4°C, then incubated in 30% surcose for 24h, and embedded in paraffin, sectioned into 5-μm-thick sections. The sections were incubated in methanol containing 3% H_2_O_2_ for 15 min and universal blocking solution for 30 min, then incubated with anti-Aβ (Servicebio, GB111197), anti-Iba1 (Servicebio, GB11105), anti-GFAP (Servicebio, GB11096), anti-CD68 (Abcam, ab125212), anti-Tau antibody (Abcam, ab32057), anti-NGF (Servicebio, GB111206) antibody at 4 °C overnight. After incubation with secondary antibody, staining was performed with DAB substrate solution.

**Western blotting**

The total protein samples were extracted using the total DNA–RNA–protein kit and the protein concentrations were measured by BCA protein assay kit. The samples were separated using SDS-PAGE (8–15% polyacrylamide gels), transferred to PVDF membranes, and incubated with primary antibodies at 4°C overnight. The following antibodies were used: Anti-Tau (phospho S396) (ab109390), Anti-Tau antibody (ab32057), anti-Aβ (Servicebio, GB111197), anti-GFAP (Servicebio, GB11096), Anti-ADAM10 (Abcam, ab124695), Anti-BACE1 (Abcam, ab108394), Anti-Nicastrin (Abcam, ab3444), Anti-β-actin antibody (Abcam, ab8226), Anti-IL1β antibody (Abcam, ab9722), Anti-TNFα (Abcam, ab6671), anti-GSK3β (Abcam, ab32391), anti-GSK3β (phospho Y216+Y279) (Abcam, ab68476), anti-TrkA (Abcam, ab86474), anti-TrkB (Servicebio, GB11295-1), anti-TrkB (phosphp Try816) (Merck, ABN1381), Anti-Caspase 3 (Cell Signaling Technology, 9662), anti-cleaved caspase 3 (Cell Signaling Technology, 9664), anti-Cd11b (Cell Signaling Technology, 49420), anti-PPARγ (Cell Signaling Technology, 2435), anti-TrkA (phosphp Try490) (Cell Signaling Technology, 9141), anti-p75NTR (Sangon Biotech, D261027), anti-Bax (Abcam, ab32503), anti-Bcl2 (Abcam, ab196495).

**Determination of Aβ levels**

Hippocampus was homogenized in lysis buffer containing a cocktail of protease inhibitors. Each sample were centrifuged at 10,000g for 15 min at 4°C. The pellet was sonicated in 70% formic acid and neutralized with 0.5 M Tris. Soluble and insoluble Aβ(1-40 and 1-42) were measured.

**Golgi staining**

Brain sections were transferred to a opaque vial containing Golgi dye solution for three days at room temperature, followed by immersion in 1% silver nitrate solution for three days.

**Statistical analysis**

Prism 8.0 software (GraphPad) was used for statistical analysis and graphical presentation. Statistical significance was determined by Student’s t test. Probability of p value <0.05 was indicated a significant difference.


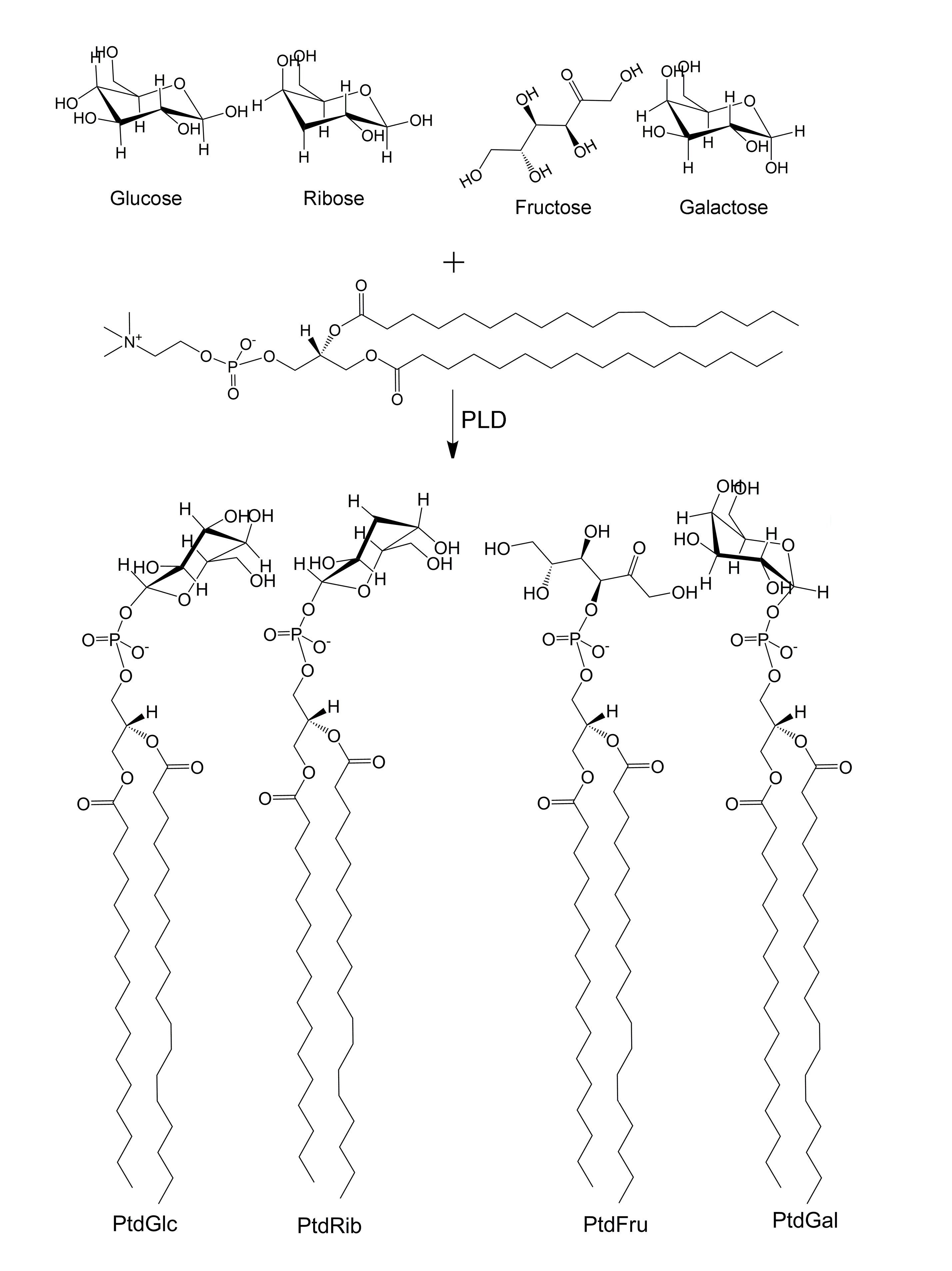


**FIGURE S1** PtdGlc and its analogues were synthesized via the approach of phospholipase D mediated transphospatidylation from soybean PtdCho.


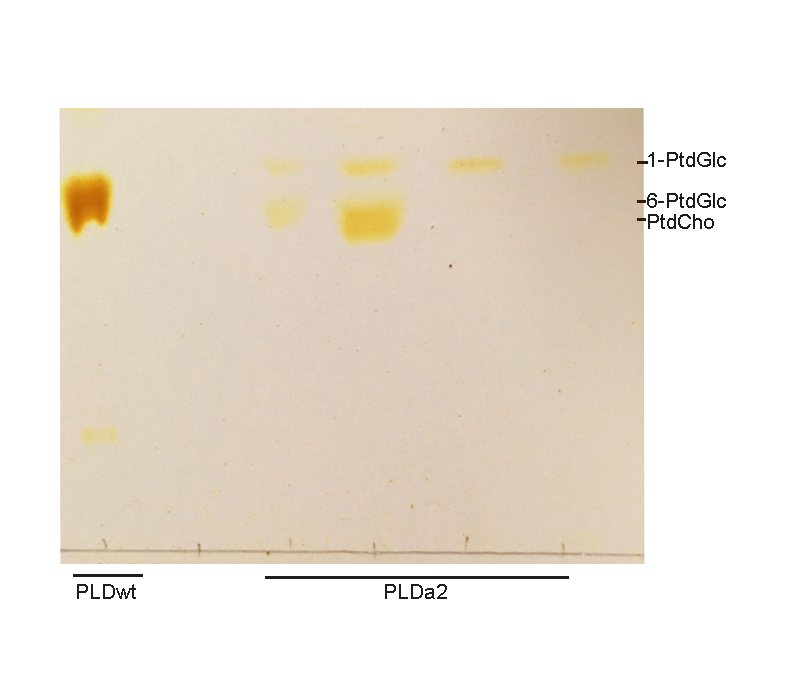


**FIGURE S2** The products after PLD mediated reaction were analyzed by TLC developed with chloroform/methanol/0.8 M NH_4_OH (75:25:3). Lane 1 PLDwt mediated reaction. The WT-PLD gave exclusively 6-PtdGlc, Lane 3 and 4 The PLDa2 mediated reaction, Lane 5 and 6 The products after purification based on the solid phase extraction (SPE).


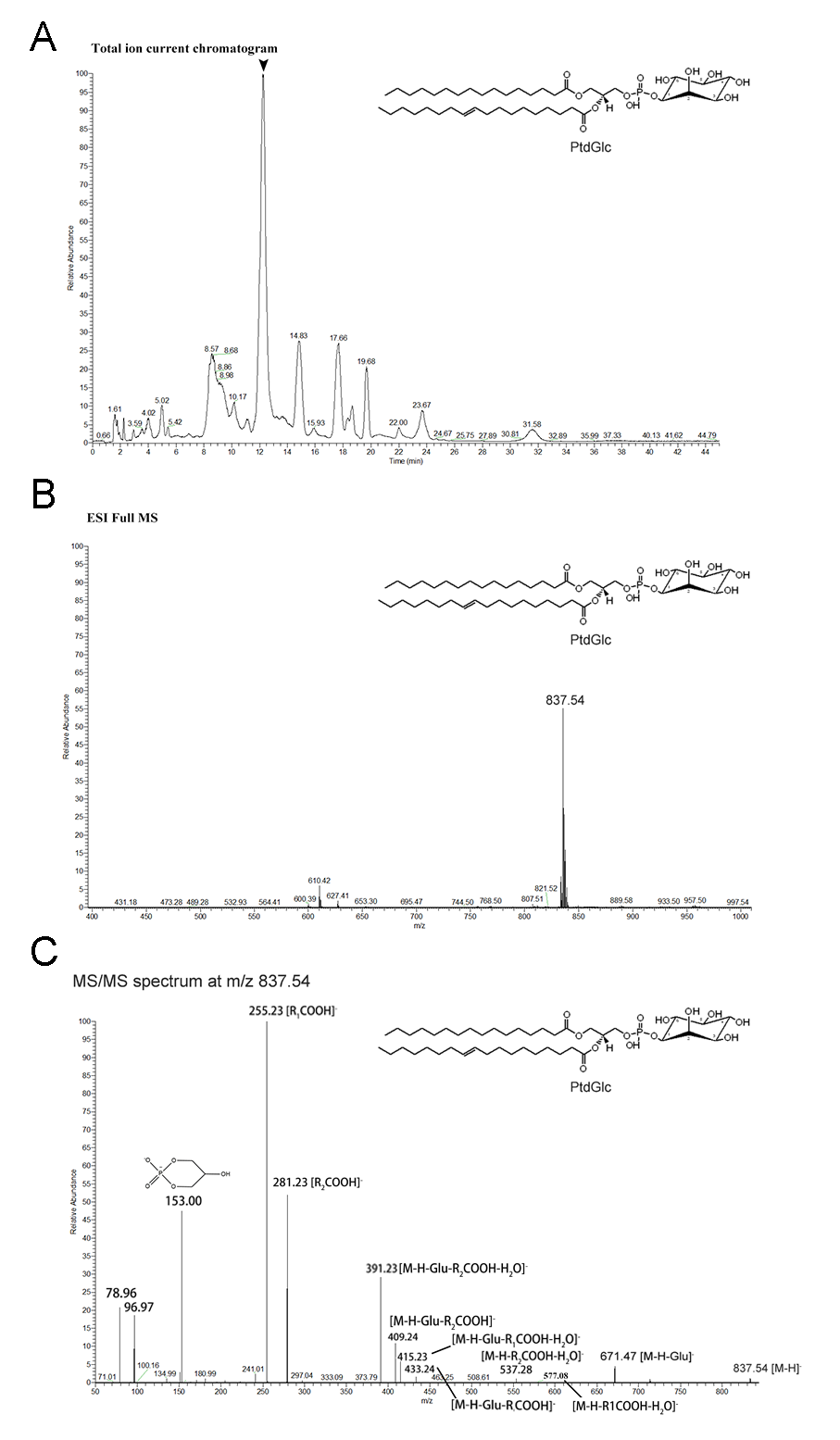


**FIGURE S3** HPLC-MS/MS analysis of PtdGlc. (A) Total ion chromatogram of PtdGlc, (B) ESI full MS of PtdGlc, (C) MS/MS spectrum at m/z 837.54.


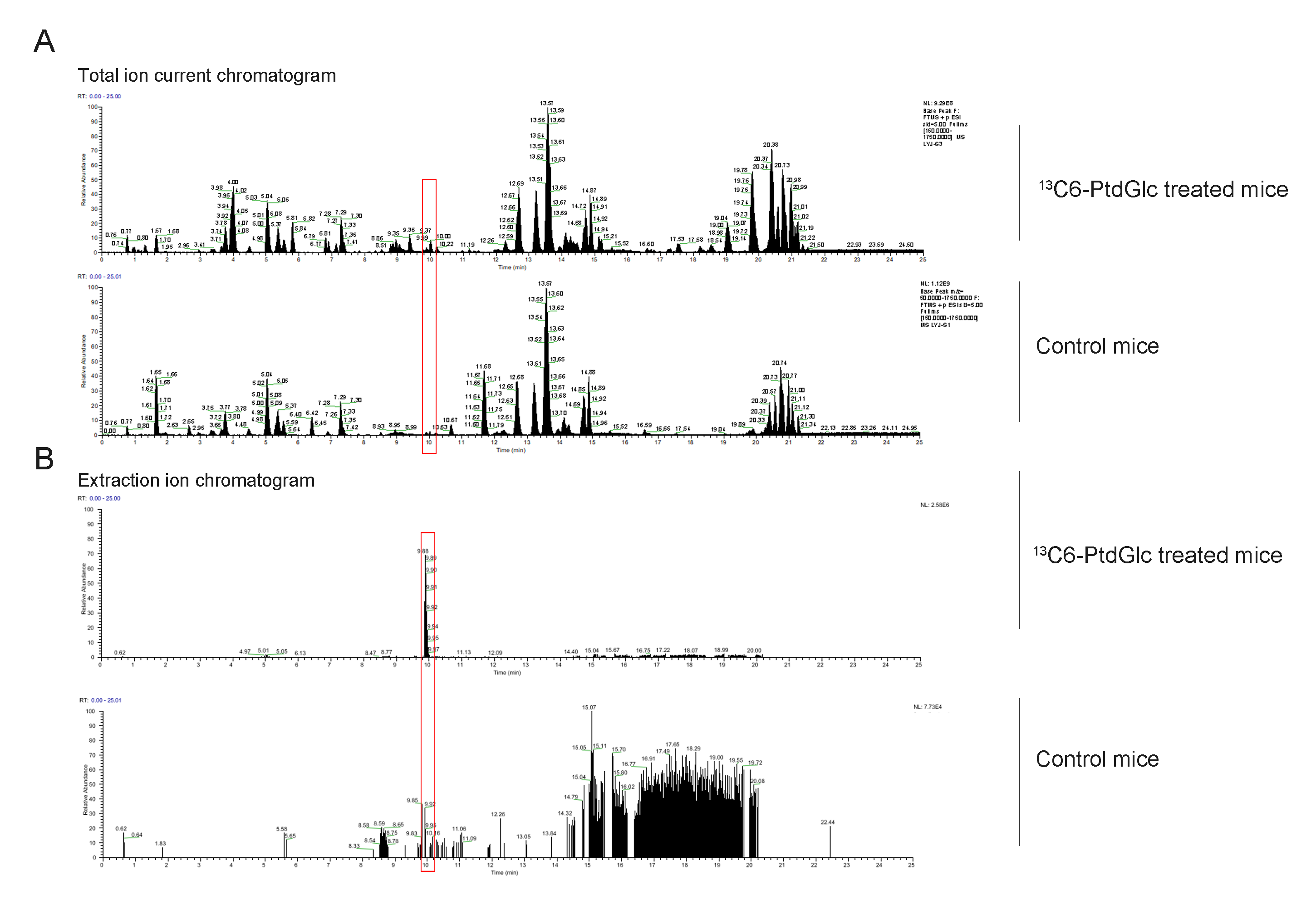


**FIGURE S4** HPLC-MS/MS analysis of ^13^C6-PtdGlc in the brain of ^13^C6-PtdGlc treated mice and Control mice. A, Total ion chromatogram of PtdGlc, B, Extraction ion chromatogram of PtdGlc.


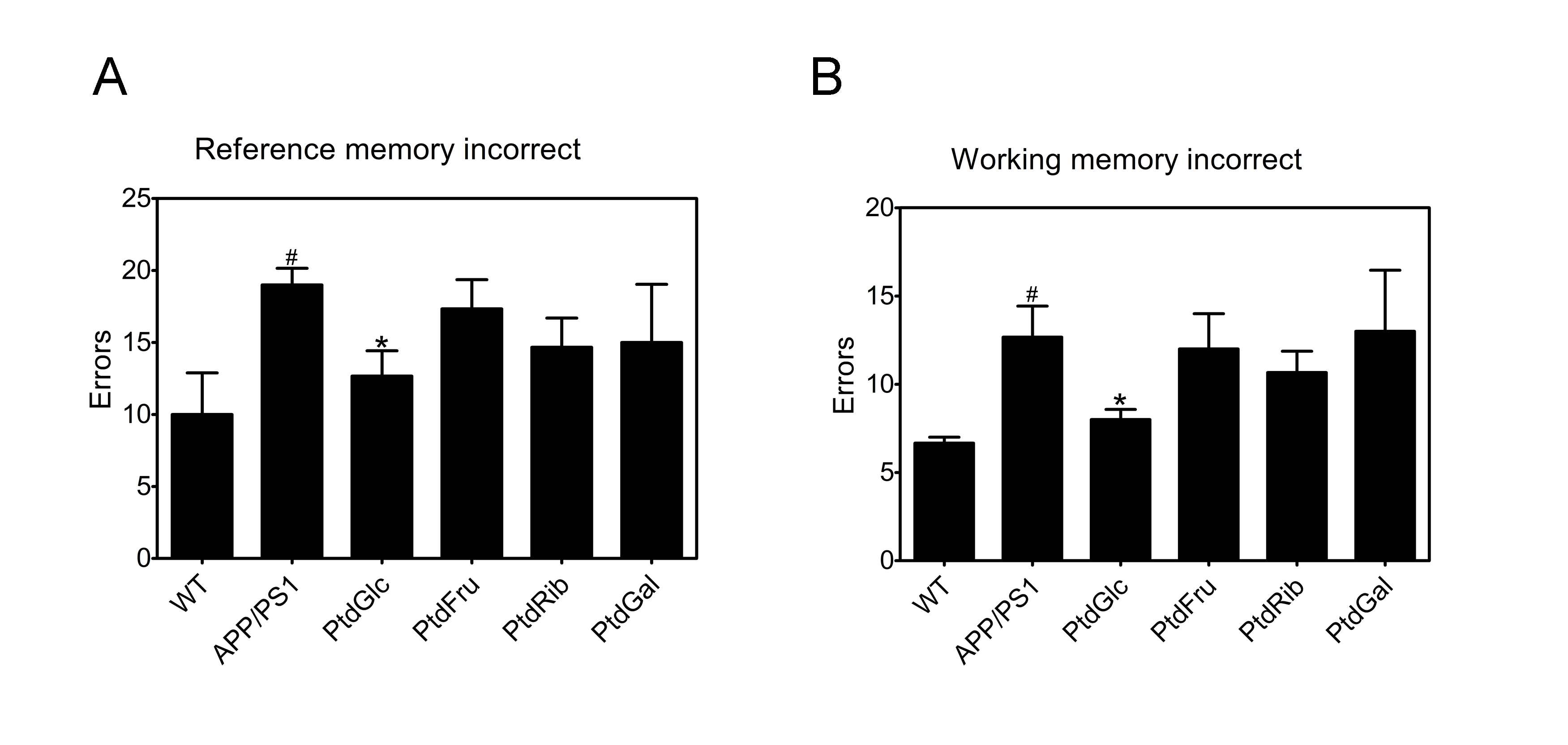
**FIGURE S5** PtdGlc rescues spatial learning and memory deficiency. (A) Number of reference memory errors in radial 8-arm maze test. (B) Number of working memory errors in radial 8-arm maze test (n=5 mice/group).


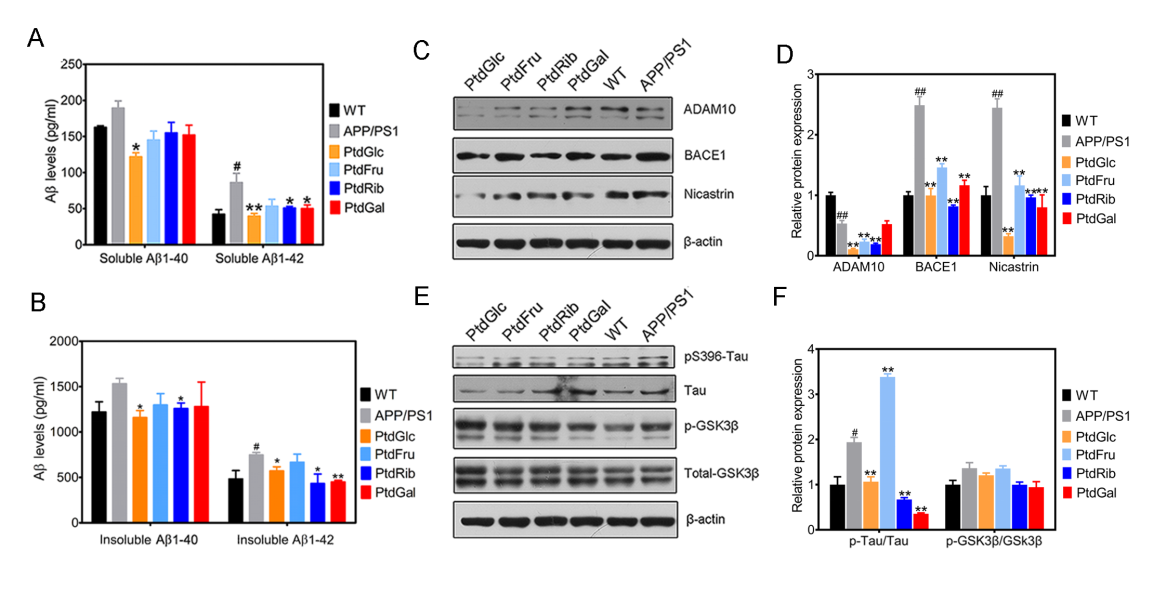


**FIGURE S6** PtdGlc attenuated Aβ and tau pathological burden (A) Quantitative analysis for the soluble forms of Aβ1-40 and Aβ1-42 in hippocampus of APP/PS1 mice (n=6 mice/group). (B) Quantitative analysis for the insoluble forms of Aβ1-40 and Aβ1-42 in hippocampus of APP/PS1 mice (n=6 mice/group). (C and D) Representative western blot of APP processing. (E and F) Representative western blot of tau and p-Tau.


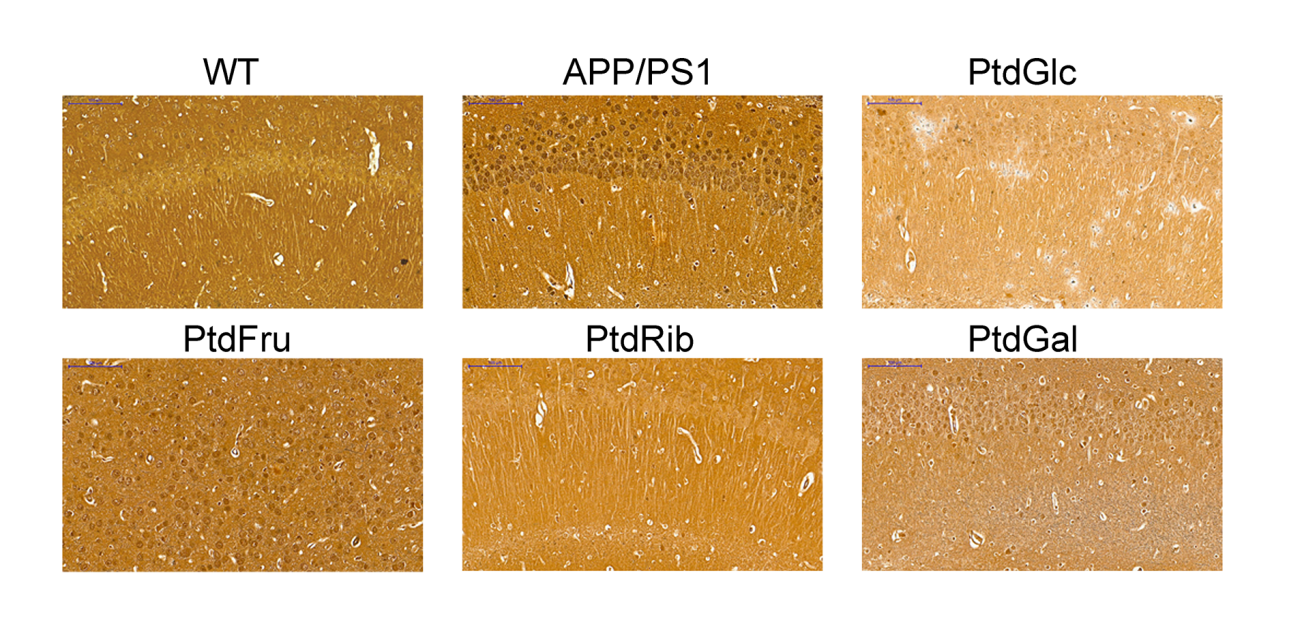


**FIGURE S7** NFTs in APP/PS1 mice were reduced with the treatment of PtdGlc, PtdRib, or PtdGal, as judged by a Bielschowsky silver staining method for detecting neurofibrillary tangles. Scale bar, 100 μm.

TABLE S1 Composition of experimental diets（g/kg）

| Ingredient | WT or APP/PS1 group | PtdGlc, PtdFru, PtdGal or PtdRib group |
| --- | --- | --- |
| Casein | 140 | 140 |
| Potato starch | 617.7 | 439.7 |
| Sucrose | 100 | 100 |
| Corn oil | 23.9 | 46 |
| Lard | 19.1 | 174 |
| Mineral mix | 35 | 35 |
| Vitamin mix | 10 | 10 |
| Cellulose | 50 | 50 |
| L-cystine | 2.5 | 2.5 |
| Choline bitartrate | 1.8 | 1.8 |
| PtdGlc, PtdFru, PtdGal or PtdRib | none added | 1 |
